# Effect of clopidogrel on the hydroxylation and sulfoxidation of omeprazole: A single dose study in healthy human volunteers

**DOI:** 10.17179/excli2016-658

**Published:** 2017-03-22

**Authors:** Lateef Ahmad, Zafar Iqbal, Shabnam Nazir, Abad Khan, Yasar Shah, Muhammad Imran Khan, Ismail Khan, Amjad Khan

**Affiliations:** 1 Department of Pharmacy, University of Swabi, Khyber Pakhtunkhwa, Pakistan; 2 Department of Pharmacy, University of Peshawar, Peshawar, Khyber-Pakhtunkhwa, Pakistan; 3 Department of Pharmacy, Kohat University of Science and Technology, Khyber Pakhtunkhwa, Pakistan; 4 Department of Pharmacy, Abdul Wali Khan University, Khyber Pakhtunkhwa, Pakistan; 5 Department of Pharmacy, Women Institute of Learning, Abbottabad; 6 Department of Pharmacy, Abasyn University, Khyber Pakhtunkhwa, Pakistan

**Keywords:** clopidogrel, omeprazole, drug interactions, pharmacokinetics, poor metabolizers, extensive metabolizers

## Abstract

Based upon the known potential interaction between omeprazole (OMP) and clopidogrel (CLOP), the current study was designed to evaluate the effect of CLOP on disposition of OMP and its two major metabolites, 5-hydroxyomeprazole (5-OH-OMP) and omeprazole sulfone (OMP-S) in healthy clinical subjects. A randomized, open label, 2-period, crossover study was designed. Twelve volunteers were selected, of whom eight were extensive metabolizers (EM) of CYP2C19 and 4 were poor metabolizers (PM). They received single dose of OMP either alone or in combination with CLOP (single dose) and samples were collected periodically to calculate various pharmacokinetic parameters. Changes in most of the pharmacokinetic parameters of OMP, 5-OH-OMP and OMP-S were insignificant (P ˃ 0.05) both in EM and PM except for the maximum concentration (C_max_) of 5-OH-OMP and OMP-S in EM. The OMP C_max _and AUC_0-∞_ was increased both in EM and PM after concomitant administration of OMP with CLOP. The 5-OH-OMP C_max _was decreased in both EM and PM, demonstrating that CLOP inhibits hydroxylation of OMP. The OMP-S C_max_ and AUC_0-∞_ were increased both in EM and PM showing that CLOP may induce sulfoxidation of OMP. It was concluded that CLOP may inhibit hydroxylation of OMP to a greater extent in EM than in PM, leading to higher OMP C_max_ and AUC_0-∞_. Furthermore, the sulfoxidation of OMP may also be induced by CLOP. So, it is suggested that both these drugs should be carefully prescribed together to avoid any harm to the patients. (Application number13/EC/Pharm. Ref number 12/Pharm).

## Introduction

Proton pump inhibitors (PPIs) are the most widely prescribed drugs for gastrointestinal tract (GIT) problems and the second most prescribed drug worldwide (Vanderhoff and Tahboub, 2002[22]). On the basis of their safety and efficacy they are prescribed excessively both in, ambulatory and admitted patients. PPIs include: omeprazole (OMP), pantoprazole, lansoprazole, rabeprazole, esomeprazole, tenatoprazole and timoprazole. Clinical studies have shown that PPIs are safe, effective and well tolerated (Reilly, 1999[16]).

PPIs perform their antisecretory action by inhibiting the proton pump, i.e., H+/K+ ATPase (adenosine triphosphatase) after conversion into its active form. These drugs undergo structural modification in the canaliculus in the presence of high acid content. More precisely, in the acidic environment, a PPI is converted from pro-drug to active form i.e., sulphenamide, which inhibits the acidic secretion by irreversibly binding to the proton pump (Stedman and Barclay, 2000[18]).

OMP is extensively metabolised in the liver. The two major metabolites of OMP are 5-hydroxy omeprazole (5-OH-OMP) (Chiba et al., 1993[6]) and omeprazole sulphone (OMP-S) (Andersson et al., 1993[2]). OMP is metabolised to thesetwo metabolites by CYP2C19 and CYP3A4, respectively. Both 5-OH-OMP and OMP-S are inactive and do not exhibitany pharmacodynamic effect (Meyer, 1996[13]). 

CLOP is an oral antiplatelet agent, belonging to the thienopyridine class. It inhibits clot formation in the blood in various diseases like peripheral vascular disease, coronary artery disease and cerebrovascular disease (Kubler et al., 2004[10]).The oral bioavailability of CLOP is less than 50 %. Absorption of CLOP is unaffected by food or antacids (McEwen et al., 1999[12]). Evidence is there to suggest that CLOP is metabolized by CYP2C19 (Fontana et al., 2007[7]) and CYP3A4 (Lau et al., 2004[11]) to its active metabolite.

There have been reports of drug-drug interaction between CLOP and PPIs. OMP diminishes CLOP effect, thus posing a threat to the patient (Norgad et al., 2009[14]; Gupta et al., 2010[8]; Kreutz et al., 2010[9]). The European and United States regulatory authorities in 2009 and 2010 warned against the concomitant use of OMP with CLOP (Angiolillo et al., 2011[3]). However, even after these recommendations co-administration of OMP with CLOP is still practiced posing threat to patients' health (Berger, 2015[4]). Likewise, OMP pharmacokinetics has also been evaluated previously showing that OMP levels are raised by lowering its metabolism through inhibition of CYP2C19 (Chen et al., 2009[5]). Considering these findings, this study was designed to evaluate the pharmacokinetics of OMP and its metabolites in presence of CLOP in both EM and PM of CYP2C19.

## Methodology

### Subjects

A group of twelve healthy volunteers were recruited for this study (divided into two groups), and written informed consent was obtained from all participants included in the study. The ages of the volunteers were in the range of 23-29 years. The body mass index was 23.22 ± 1.16 lb/in^2^. Electrocardiogram, routine lab tests including hematological, kidney and liver function tests were performed for all the volunteers. They were physically examined and their medical history was also evaluated. All volunteers were non-smokers and abstained from taking any other medicine and juices in the week prior to the study. Volunteers were served standard breakfast and lunch during the study. Volunteers were divided into extensive metabolizers (n = 8) and poor metabolizers (n = 4) of CYP2C19 on the basis of drug-metabolite ratio (OMP: 5-OH-OMP).

### Study design

This study was conducted in accordance to “World Medical Association (WMA) declaration of Helsinki−*Ethical principles for medical research involving human subjects*” and its amendments. The study was approved by the ethical committee, Department of Pharmacy, University of Peshawar, Pakistan. (Application number13/EC/Pharm. Ref number 12/Pharm). It was a single dose, 2-period, 2-sequence study with 14 days washout period. OMP (Omega 40 mg, Ferozsons Labs Pvt., Ltd., Nowshehra, Pakistan) was either administered alone or in combination with CLOP (Clopid 150 mg, Ferozsons Labs Pvt., Ltd., Nowshehra, Pakistan) as shown in Table 1(Tab. 1).

### Analytical method and pharmacokinetic analysis

Approximately 3 mL of blood was collected from each volunteer at 0, 0.5, 1, 2, 3, 4, 6, and 8 hr after dose administration in heparinized tubes. After centrifugation, the plasma was transferred into properly labeled eppendroff tubes and stored at a temperature of -20 °C until analysis. OMP and its metabolites were extracted from the plasma using precipitation method and were analyzed using LCMS method. The mobile phase consisted of 0.1 % formic acid in acetonitrile: 0.1 % formic acid in water (40/60 v/v) using Hichrom RP18 (150 × 3.0 mm, 3 µm UK) as a stationary phase interfaced with a LTQ Orbit rap mass spectrometer. Pantoprazole was used as an internal standard (Ahmad et al., 2015[1]).

**Figure 1:**
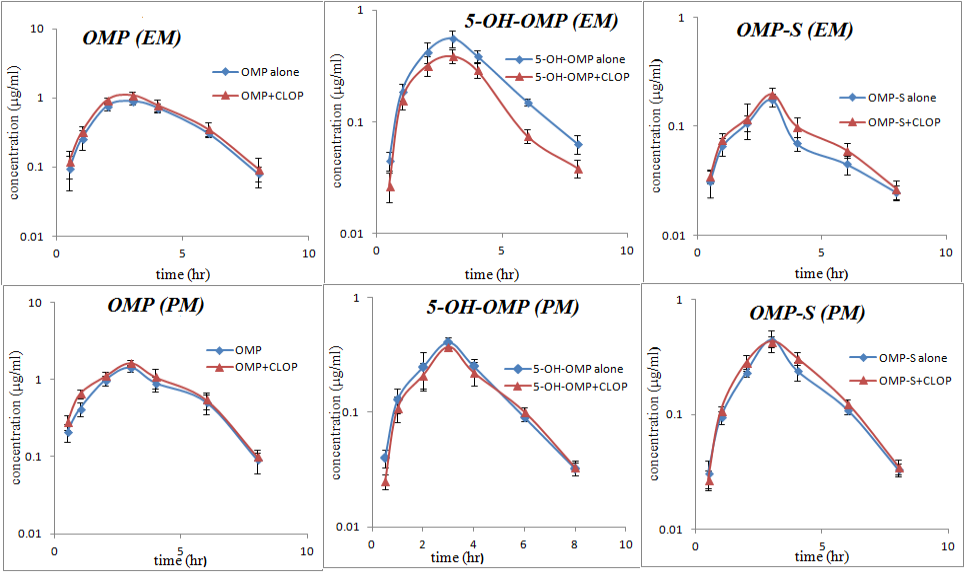
Mean ± SD plasma concentration (µg/ml) of OMP, 5-OH-OMP and OMP as a function of time in EM (n = 8) and PM (n = 4), when OMP (40 mg) administered alone and in combination with CLOP (150 mg).

The plasma concentration (mean ± SD) as a function of time curves for OMP, 5-OH-OMP and OMP-S were plotted when OMP was administered alone or in combination with CLOP as shown in Figure 1(Fig. 1). 

Various PK parameters were calculated using the PK Solutions software. The maximum plasma concentration (C_max_) and time to reach C_max _(t_max_) were determined from plasma concentration-time profile of OMP, 5-OH-OMP and OMP-S. The AUC_0-∞_ was determined by trapezoidal rule. The elimination half life was determined by using first-order equation, i.e.,


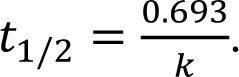


The apparent oral clearance (CL/F) was determined by using equation, Dose/AUC_0-∞_. The pharmacokinetic data was interpreted statistically using SPSS software (version 16.0; SPSS, Inc, Chicago, IL). A paired t-test was performed for the comparison of pharmacokinetic parameters of OMP, 5-OH-OMP and OMP-S when OMP was administered alone and in combination with CLOP in both EM and PM. P < 0.05 was considered statistically significant.

## Results

Volunteers were divided into EM and PM by drug metabolite ratio (Shilbayeh and Tutunji, 2006[17]), i.e., [AUC]_OMP/5-OH-OMP_ for EM was 1.94 ±0.602 while for PM it was 9.84 ± 4.543. [AUC]_OMP/OMP-S_ for EM and PM was 13.64 ± 2.711 and 9.85 ± 4.98, respectively.

The pharmacokinetic parameters for both OMP, 5-OH-OMP and OMP-S alone and in combination with CLOP in EM and PM are shown in Table 2(Tab. 2) and Table 3(Tab. 3), respectively.

Plots for plasma concentration as a function of time were constructed for OMP, 5-OH-OMP and OMP-S when the subjects received OMP alone or in combination with CLOP in EM and PM as shown in Figure 1(Fig. 1).

The changes in all the parameters of OMP and its metabolites were insignificant after administration of CLOP except the C_max_ of 5-OH-OMP and OMP-S in EM which was significantly altered (P < 0.05). The AUC_0-∞_ and C_max_ of OMP were increased in both EM and PM after concomitant administration of CLOP but the percent increase is much more in EM than PM. The AUC_0-∞_ and C_max_ of 5-OH-OMP were decreased in both EM and PM after co-administration of CLOP with OMP. An increase in the C_max_ and AUC_0-∞_in the other metabolite formed through CYP3A4 (OMP-S) was observed both in EM and PM.

## Discussion

In literature, only one report was found that supported the interaction between OMP and CLOP. This previously reported study illustrates that the interaction between OMP and CLOP is based on the inhibition of CYP2C19 by CLOP (Chen et al., 2009[5]). Changes in pharmacokinetic parameters of OMP and its metabolites were observed in the present study for both EM and PM. The increase in the C_max_ (8.25% ± 3.3 %) and AUC_0-∞_ (9.33% ± 5.13%) of OMP after concomitant administration of CLOP in EM may be due to the inhibition of CYP2C19, which resulted in decreased formation of 5-OH-OMP**. **CLOP is also a substrate of CYP3A4 but there is no study to date that suggests whether it is inducer or inhibitor of CYP3A4. Drugs that are substrates of CYP450, may also act as inhibitor or inducer of that particular CYP isoform. For example, carbamazepine is a substrate as well as inducer of CYP3A4, similarly clarithromycin is a substrate as well as inhibitor of CYP3A4 (Zhou, 2008[24]). Though OMP is not mainly metabolized by CYP3A4 but a slight increase in AUC_0-∞_ and C_max_ of OMP-S after concomitant in EM may be due to the induction of CYP3A4 by CLOP. The other possible reason for increase of OMP-S may be the CLOP induced inhibition of CYP2C19 dependent hydroxylation, and in turn OMP is predominantly metabolized by CYP3A4.

Previously reported studies of OMP pharmacokinetics suggest that OMP concentration (C_max_ and AUC_0-∞_) was greater in PM as compared to EM. On the other hand, 5-OH-OMP concentration was high in EM and low in PM, and OMP-S concentration was reported to be high in PM as compared to EM, suggesting that sulfoxidation is the major metabolic pathway in PM (Uno et al., 2007[21]; Yin et al., 2004[23]). In present study, the OMP concentration was increased in PM and 5-OH-OMP concentration was decreased after co-administration of CLOP while OMP-S was increased in PM after concomitant administration of OMP with CLOP. The decrease in hydroxylation or increase in sulfoxidation of OMP in PM was insignificant and the changes in the pharmacokinetic parameters were much smaller by considering the percent decrease or increase in their respective concentration as shown in table 3(Tab. 3). CLOP absorption and its active metabolite formation are decreased by the P-glycoprotein efflux (Taubert et al., 2006[20]). Similarly OMP is a substrate as well as inhibitor of P-glycoprotein (Pauli-Magnus et al., 2001[15]). The other possible reason for the increase of OMP concentration may be inhibition of this efflux pump by CLOP but this is a weak mechanistic approach as previously it has been reported that OMP pharmacokinetics were not altered by fexofenadine, a P-glycoprotein inhibitor/substrate (Takahata et al., 2004[19]).

In conclusion, CLOP inhibits hydroxylation of OMP to a greater extent in EM than in PM, leading to higher OMP C_max_ and AUC_0-∞_. Furthermore, the sulfoxidation of OMP is induced either by induction of CYP3A4 or by inhibition of CYP2C19 as result OMP is metabolized to a greater extent by CYP3A4. It is suggested that OMP and CLOP should not be administered concomitantly.

## Acknowledgement

Authors are thankful to University of Peshawar for providing the vicinity and facilities to conduct the experiments.

## Conflict of interest

Conflict of interest doesn’t exist.

## Figures and Tables

**Table 1:**
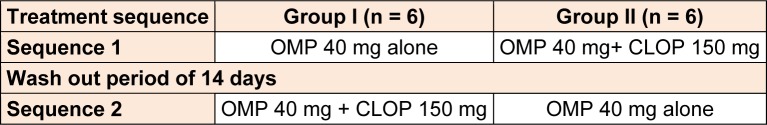
The study design of drug-drug interaction of omeprazole with clopidogrel in healthy human volunteers

**Table 2:**
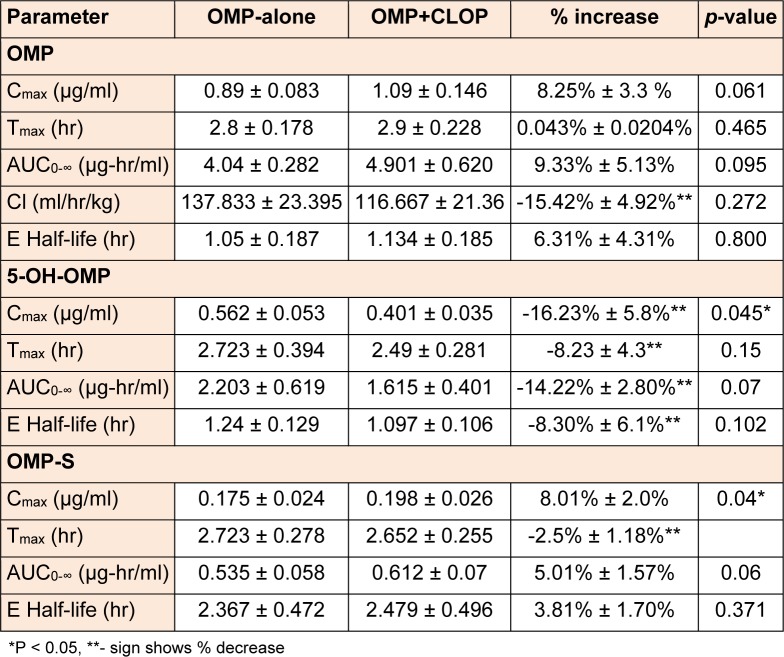
Pharmacokinetic parameters of omeprazole, 5-hydroxyomeprazole and omeprazole sulfone in extensive metabolizers when the subjects received omeprazole (40 mg) alone and in combination with clopidogrel(150 mg)

**Table 3:**
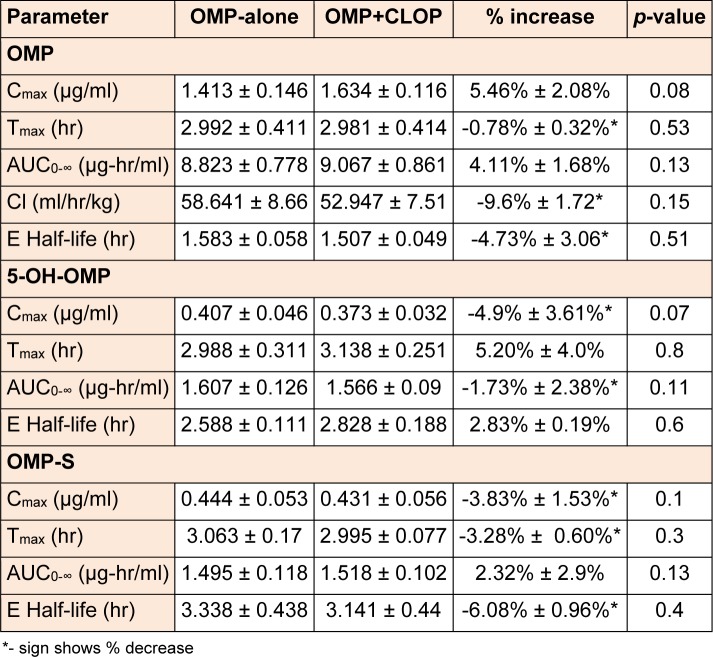
Pharmacokinetic parameters of omeprazole, 5-hydroxyomeprazole and omeprazole sulfone in poor metabolizers when the subjects received omeprazole (40 mg) alone and in combination with clopidogrel (150 mg).
